# Socioeconomic Position and Malnutrition among Older Adults: Results from the FRADEA Study

**DOI:** 10.1007/s12603-018-1061-1

**Published:** 2018-06-26

**Authors:** Emiel O. Hoogendijk, T. Flores Ruano, M. MartÃ­nez-Reig, M. LÃ³pez-Utiel, S. Lozoya-Moreno, E. Dent, P. Abizanda

**Affiliations:** 1 Department of Epidemiology & Biostatistics, Amsterdam Public Health research institute, VU University medical center, Amsterdam, the Netherlands; 2 Department of Geriatrics, Complejo Hospitalario Universitario of Albacete, Albacete, Spain; 3 CIBERFES, Ministerio de EconomÃ­a y Competitividad, Madrid, Spain; 4 Torrens University Australia, Adelaide, Australia; 5 Baker Heart and Diabetes Institute, Melbourne, Australia

**Keywords:** Older adults, malnutrition, nutritional assessment, socioeconomic position

## Abstract

**Objectives:**

Low socioeconomic position (SEP) is related to many health-related conditions in older adults. However, there is a lack of knowledge on the association between SEP and malnutrition, a condition with serious consequences for older people in terms of quality of life and adverse health events. In the current study, we investigated socioeconomic inequalities in malnutrition and sub-domains of malnutrition in a sample of Spanish older adults.

**Design:**

Cross-sectional population-based study.

**Setting:**

Urban area of Albacete, Spain. Participants: 836 participants over age 70 from the first measurement wave (2007-2009) of the Frailty and Dependence in Albacete (FRADEA) study, a population-based cohort study.

**Measurements:**

Educational level and occupational level were the indicators of SEP. Nutritional risk was measured with the Mini Nutrition AssessmentÂ® Short Form (MNAÂ®-SF). Logistic regression analyses were performed.

**Results:**

For both socioeconomic indicators there was a statistically significant association with nutritional risk (OR low education=1.99, 95% CI=1.18-3.35; OR low occupational level=1.71, 95% CI=1.08-2.72). However, these associations disappeared after adjusting for age and sex (OR low education=1.51, 95% CI=0.88-2.60; OR low occupational level=1.32, 95% CI=0.80-2.17). In adjusted models, statistically significant associations between SEP and sub-domains of the MNAÂ®-SF were observed, but these associations were not consistent across socioeconomic indicators.

**Conclusions:**

This study found that malnutrition is a condition that can appear in any older adult, regardless of their socioeconomic group. These findings suggest that interventions to prevent malnutrition in older adults can be targeted at a general older population, and do not have to be SEP specific.

## Introduction

Older people with a low socioeconomic position (SEP) have a higher risk of various geriatric conditions, and adverse outcomes related to these conditions. This includes, for example, frailty and polypharmacy, which have shown to be more prevalent in older adults with low education or low levels of income (1, 2, 3). Another geriatric condition with serious consequences for older people in terms of quality of life and adverse health events is malnutrition (4, 5). However, for malnutrition the association with SEP is less well established.

Malnutrition in older adults is being increasingly recognized as a major contributor to early mortality and functional decline (6), with around 5% of community-dwelling older adults affected, and an additional 30% at risk of developing the condition (7). There are many well-known risk factors for malnutrition in older people, including swallowing difficulties, early satiation, loss of appetite and multimorbidity (8, 9). What is less clear is the association of SEP with malnutrition. Whilst malnutrition is often linked with low education and poverty, the majority of studies supporting this hypothesis are either conducted in developing countries, among children, or in older adults residing in residential care (10, 11, 12, 13). The few studies that have been conducted among communitydwelling older adults show mixed results. For instance, an Italian study found an association between low educational level and nutritional risk (14), yet, on the other hand, a recent Polish study concluded that low educational level was not an independent risk factor of malnutrition (15). Both studies made use of the Mini Nutritional AssessmentÂ® Short Form (MNAÂ®-SF), a well-validated multidimensional measurement instrument (16), and the recommended instrument by the European Society of Clinical Nutrition (ESPEN) for malnutrition screening of community-dwelling older people (17). Despite the comprehensive nature of these studies, they did not further investigate socioeconomic inequalities in the sub-domains of the MNAÂ®-SF. It could be that, even when there is no consistency in in the overall association between SEP and malnutrition, there may still be a clear SEP pattern for the sub-domains of the MNAÂ®-SF. These sub-domains include decreased food intake, weight loss, mobility limitations, acute health status (psychological stress/ acute disease), neuropsychological issues (dementia and/or depression) and low body mass index (BMI) â€“ the latter of which can be substituted for calf circumference measurement (16). Knowledge of the relationship between SEP and these sub-domains of malnutrition will build evidence regarding nutritional care for older people, and better position healthcare practitioners to identify and manage malnutrition.

The objective of the current study was to investigate socioeconomic inequalities in malnutrition and sub-domains of malnutrition among older adults, using data from the Frailty and Dependence in Albacete (FRADEA) study, a sample of Spanish community-dwelling older adults. Previous work on malnutrition in the same cohort showed that about 30% of this population was malnourished or at risk of malnutrition, as determined by the MNAÂ®-SF (4).

## Methods

### Design and study population

Cross-sectional data from the first wave (2007-2009) of the FRADEA study were used. FRADEA is a populationbased cohort study among older adults aged 70 and over in the urban area of Albacete in Spain. Details on the methods and characteristics of the study sample have been published before (18). In brief, to obtain a representative sample of a Spanish urban older population, 1172 people aged 70 and over were randomly selected from registered health care holders in the city of Albacete in 2007 (n = 18137), of which 993 individuals (84.7%) agreed to participate. Data were collected by faceto- face interviews and clinical measurements at the geriatrics department of the Complejo Hospitalario Universitario in Albacete. This was done by four trained nurses between November 2007 and November 2009. Of the 993 participants included in the FRADEA study, 157 (15.6%) had no valid data on malnutrition. This resulted in a final sample of 836 people included in the current analysis. The FRADEA study received approval by the Albacete health region Independent Ethics Committee and the Albacete University Hospital Ethics Committee. All participants provided signed informed consent.

### Measurements

Our SEP measures included educational level and occupational level. Respondents were asked about their highest level of education. There were five answering categories: illiterate, primary school not completed, primary school completed, secondary school, and university (18). Respondents were also asked about their main occupation during their working life. The Spanish National Classification of Occupations was used to differentiate occupational levels (19). Seven levels were distinguished (inadequately described occupations, unskilled occupations, partly skilled occupations, manual skilled occupations, non-manual skilled occupations, intermediate occupations, and professional occupations). Due to skewed distributions we dichotomized both socioeconomic indicators. Those with primary school or less were distinguished from people with secondary school or more to indicate low and high level of education. Inadequately described occupations, unskilled occupations, partly skilled occupations, and manual skilled occupations were considered as low occupational level, while non-manual skilled occupations, intermediate occupations, and professional occupations were considered as high occupational level.

Nutritional status was measured with the MNAÂ®-SF (16). This instrument includes six items which evaluate decreased food intake, weight loss, mobility, acute health status, neuropsychological problems, and anthropometric data (BMI). The summed score of the MNAÂ®-SF ranges between 0 and 14. In the current study, nutritional risk was used as the main outcome measure. This includes all participants who are malnourished or at risk of malnutrition, determined by the recommended cut-off of <12 points on the MNAÂ®-SF (4). Subdomains of malnutrition were dichotomized. Decreased food intake was considered present if respondents had a moderate to severe decrease in food intake in the past 3 months due to loss of appetite, digestive problems, or chewing/swallowing difficulties. Weight loss was present if respondents had any weight loss in the past 3 months. Limited mobility meant that an older adult was bed or chair bound, or was able to get out of bed/chair but never goes out. To measure acute disease or psychological stress, respondents were asked whether they suffered from psychological stress or an acute disease in the past 3 months (yes/no). Neuropsychological problems were present if a respondent had dementia and/or depression. Finally, low BMI was defined as a BMI of lower than 21 kg/m2 (20).

Other variables to characterize the current study sample included age, sex, Barthel Index, Charlson index, Mini-Mental State Examination (MMSE), and frailty. The Barthel Index provides insight into basal functional status. It is a score from 0 to 100, where lower scores indicate reduced ability to perform basic activities of daily living, such bathing, grooming, dressing, eating, toilet use, continence, and mobility (21). The presence of comorbidity was summarized with the Charlson comorbidity index (22). Cognitive functioning was measured with the MMSE (range 0â€“30, with higher scores indicating better cognitive functioning) (23). Frailty was assessed with the criteria of Fried's frailty phenotype (24). Respondents were considered to be frail if at least three out of five criteria were present: weight loss, slow gait speed, low grip strength, exhaustion, and low physical activity, as described in more detail elsewhere (25).

### Statistical analysis

Descriptive analyses were performed to outline details of the study sample. To compare characteristics of participants with a low educational/occupational level with those with a high educational/occupational level, t-tests for continuous variables and chi-square tests for categorical variables were performed respectively. Socioeconomic inequalities in malnutrition were determined using logistic regression analyses, with nutritional risk as outcome measure. For both socioeconomic indicators, two regression models were performed: (i) a univariate model without adjustment for covariates; and (ii) a model adjusted for age and sex. These regression analyses were repeated for each MNAÂ®-SF sub-domain. All analyses were done in IBM SPSS Statistics 22 (IBM Corp. Armonk, NY).

## Results

Malnutrition or the risk of malnutrition was present in 30.3% of the study population. For the MNAÂ®-SF sub-domains, the highest prevalence was observed for limited mobility and neuropsychological problems. Table 1 also shows differences in characteristics by SEP. The prevalence of malnutrition and the risk for malnutrition was lower among higher educated older adults (19% among higher educated versus 31.8% among lower educated). Three sub-domains of malnutrition were more prevalent in lower educated people (weight loss, limited mobility, and neuropsychological problems). Furthermore, respondents with a higher educational level were younger, more often male, and had a better functional and cognitive status. Nutritional status also differed by occupational level (21.3% with nutritional risk or malnutrition among older adults with a high occupational level versus 31.7% among those with a low occupational level). The MNAÂ®-SF sub-domains weight loss, limited mobility, and acute disease or psychological stress were more prevalent among those with a low occupational level, while low BMI was more often present in people with a high occupational level. Respondents with a higher occupational level were also more often male, and had a better cognitive status.

**Table 1:** Characteristics of the study sample for the total population and by socioeconomic position

	**Total**		**By educational level**			**By occupational level**		
	**(N = 836)**		**Low (N=736)**	**High (N=100)**		**Low (N=707)**	**High (N=122)**	
	**N**	**% or M (SD)**	**% or M (SD)**	**% or M (SD)**	p*	**% or M (SD)**	**% or M (SD)**	p*
Age, years	836	78.5 (5.9)	78.7 (5.9)	77.2 (5.9)	0.02	78.6 (5.8)	78.1 (6.5)	0.36
Sex								
Female	492	58.9	71.0	29.0	<0.001	65.2	20.5	<0.001
Male	344	41.1	37.1	62.9		34.8	79.5	
Barthel index	831	89.2 (17.8)	88.7 (18.2)	93.3 (14.0)	<0.01	88.9 (17.8)	91.8 (16.5)	0.09
Charlson comorbidity index	835	1.2 (1.4)	1.2 (1.4)	1.1 (1.7)	0.66	1.2 (1.4)	1.1 (1.4)	0.63
MMSE score	734	24.9 (4.8)	24.6 (4.9)	26.8 (3.2)	<0.001	24.6 (4.9)	26.7 (3.6)	<0.001
Frailty, % yes	159	19.3	20.1	14.0	0.15	19.9	16.5	0.39
MNAÂ®-SF								
Normal (12-14)	583	69.7	68.2	81.0	0.02	68.3	78.7	0.02
At risk of malnutrition (8-11)	222	26.6	27.7	18.0		28.3	16.4	
Malnourished (0-7)	31	3.7	4.1	1.0		3.4	4.9	
MNAÂ®-SF sub-domains								
Decreased food intake	137	16.4	17.1	11.0	0.12	16.8	14.8	0.57
Weight loss	166	21.0	21.9	14.1	0.08	22.5	12.8	0.02
Limited mobility	194	23.2	25.0	10.0	<0.01	24.5	14.8	0.02
Acute disease or psychological stress	178	21.3	21.4	21.0	0.93	22.6	13.9	0.03
Neuropsychological problems	194	23.2	24.9	11.0	<0.01	24.1	16.4	0.06
Low BMI	32	4.1	3.8	6.0	0.31	3.1	9.9	<0.001

* t-test or chi-square test

Tables 2 and 3 present the results of the logistic regression analyses. For both educational level and occupational level socioeconomic inequalities in nutritional risk were observed in the unadjusted model, with a higher risk for malnutrition among lower socioeconomic groups (OR = 1.99, 95% CI = 1.18-3.35, and OR = 1.71, 95% CI = 1.08-2.27, respectively). After adjusting for age and sex, the effects of educational level and occupational level were no longer statistically significant (OR = 1.51, 95% CI = 0.88-2.60, and OR = 1.32, 95% CI = 0.80- 2.17, respectively). Since the SEP effects were not statistically significant anymore after controlling for age and sex, no further adjustment for other covariates was applied.

**Table 2:** Associations of educational level with nutritional risk and MNAÂ®-SF sub-domains

	**Crude effect**			**Adjusted for age, sex**		
	**Low education OR (95% CI)**	**High education (ref.) OR (95% CI)**	**p**	**Low education OR (95% CI)**	**High education (ref.) OR (95% CI)**	**p**
Total score						
MNAÂ®-SF <12	1.99 (1.18-3.35)	1.00	0.01	1.51 (0.88-2.60)	1.00	0.14
Sub-domains						
Decreased food intake	1.67 (0.87-3.22)	1.00	0.12	1.20 (0.60-2.37)	1.00	0.61
Weight loss	1.71 (0.94-3.09)	1.00	0.08	1.45 (0.79-2.66)	1.00	0.23
Limited mobility	3.00 (1.53-5.89)	1.00	<0.01	2.47 (1.16-5.28)	1.00	0.02
Acute disease or						
psychological stress	1.02 (0.61-1.71)	1.00	0.93	1.12 (0.66-1.89)	1.00	0.68
Neuropsychological						
problems	2.68 (1.40-5.13)	1.00	<0.01	2.11 (1.09-4.10)	1.00	0.03
Low BMI	0.62 (0.25-1.55)	1.00	0.31	0.44 (0.16-1.22)	1.00	0.12

OR = Odds Ratio; 95% CI = 95% Confidence Interval

**Table 3:** Associations of occupational level with nutritional risk and MNAÂ®-SF sub-domains

		**Crude effect**			**Adjusted for age, sex**	
	**Low occupational level**	**High occupational level (ref.)**		**Low occupational level**	**High occupational level (ref.)**	
	**OR (95% CI)**	**OR (95% CI)**	**p**	**OR (95% CI)**	**OR (95% CI)**	**p**
Total score						
MNAÂ®-SF <12	1.71 (1.08-2.72)	1.00	0.02	1.32 (0.80-2.17)	1.00	0.28
Sub-domains						
Decreased food intake	1.17 (0.68-2.00)	1.00	0.57	0.80 (0.44-1.45)	1.00	0.46
Weight loss	1.97 (1.11-3.49)	1.00	0.02	1.65 (0.91-3.00)	1.00	0.10
Limited mobility	1.87 (1.10-3.18)	1.00	0.02	1.81 (0.96-3.43)	1.00	0.07
Acute disease or						
psychological stress	1.80 (1.05-3.10)	1.00	0.03	2.08 (1.18-3.66)	1.00	0.01
Neuropsychological problems	1.62 (0.97-2.69)	1.00	0.06	1.13 (0.66-1.93)	1.00	0.67
Low BMI	0.29 (0.14-0.60)	1.00	<0.01	0.18 (0.07-0.46)	1.00	<0.001

OR = Odds Ratio; 95% CI = 95% Confidence Interval

There were also statistically significant associations between SEP and sub-domains of the MNAÂ®-SF (Tables 2 and 3). In adjusted models, limited mobility (OR = 2.47, 95% CI = 1.16- 5.28) and neuropsychological problems (OR = 2.11, 95% CI = 1.09-4.10) were more often observed among lower educated people. For the other indicator of SEP, occupational level, statistically significant associations were present for acute disease/psychological stress and low BMI. Acute disease/ psychological stress was more often present among those with a lower occupational level compared to those with a high occupational level (OR = 2.08, 95% CI = 1.18-3.66), while a low BMI was less common in those with a low occupational level (OR = 0.18, 95% CI = 0.07-0.46).

## Discussion

socioeconomic inequalities in malnutrition and sub-domains of malnutrition among community-dwelling older adults. Results from adjusted models indicated that low levels of either education or occupation were not associated with the overall risk for malnutrition, as determined by the MNAÂ®- SF. Associations between SEP and sub-domains of the MNAÂ®-SF were observed, but these associations were not consistent across SEP indicators: educational level was related to limited mobility and neuropsychological problems, whereas occupational level was associated with acute disease/ psychological stress and low BMI.

This study adds to existing evidence showing that the relationship between socioeconomic indicators and being at risk for malnutrition is inconsistent. We found that educational level was not associated with malnutrition when adjusted for covariates. This finding is in line with a previous study among older community-dwelling adults in Poland (15), although disagrees with a study among older Italians in which a higher risk for malnutrition was associated with lower education level (14). However, this same Italian study found no evidence for the link between occupational level or financial conditions and the risk of malnutrition. In future research, the role of income for nutritional risk may be studied in greater detail, as income may be related to differences in nutrient intake (i.e., insufficient quality and quantity of food intake), an underlying cause of poor nutritional status (26). The current study lacked information on income, and previous studies used only proxy measures for income (e.g., self-reported poverty) (15).

There was no clear pattern in the associations between SEP and sub-domains of the MNAÂ®-SF. Limited mobility and neuropsychological problems were more prevalent among lower educated people, and acute disease/psychological distress was more common in those with a lower occupational level. Educational inequalities in mobility and neuropsychological problems have been observed before, as well as income differences in diseases (27, 28, 29). However, we would have expected these associations to be more consistent across SEP indicators, as shown in previous studies on socioeconomic inequalities in morbidity and disability (29). Surprisingly, our results showed a higher prevalence of low BMI among people with a higher occupational level. This may be explained by the low prevalence of low BMI, which limits the reliability of the analysis on this sub-domain, and by the fact that obesity not low BMI is usually more prevalent among older adults with a low SEP (27,29).

Whilst there is a paucity of literature investigating the relationship between SEP and malnutrition, the opposite is true of the geriatric condition of frailty. Multiple studies of frailty and its link to SEP have been conducted across various settings of older adults (27,30-34). Moreover, to our knowledge, frailty has been consistently found to associate with SEP in all studies to date. Given that frailty is a common manifestation of undernutrition, we expected to find a similar relationship between SEP and malnutrition. The lack of such a relationship in our study (and that of the Polish study mentioned above (15)) was surprising and suggests that other domains of malnutrition (low physical activity, depression, lack of appetite, inadequate care and support, poor oral health amongst others) may have much more of an impact of malnutrition development than low SEP, highlighting the multifactorial nature of malnutrition.

Strengths of the present study include the large study sample size with a high cooperation rate (84.7%), the comprehensive dataset with multiple indicators of SEP, and the use of the well-validated MNAÂ®-SF to indicate nutritional status. Despite these strengths, the study did have its limitations, including its cross-sectional design. Accordingly, results from this study do not infer causation. Furthermore, we used multiple sources of socioeconomic data to measure SEP, as recommended in older populations (35). However, unfortunately, information on income is lacking in the FRADEA study, to further complement the socioeconomic data. Another limitation may be the skewed distributions of both socioeconomic indicators, with a large proportion of the respondents that belong to the low SEP groups. However, this reflects the characteristics of the 70+ population in Spain, where many older adults of this generation started to work at early age, instead of going to high school or university.

Our study has implications for managing and intervening on malnutrition in daily practice. Since malnutrition has serious consequences for health and functioning of older adults, it is important to develop intervention strategies aimed at preventing or reducing malnutrition. This may lead to improved outcomes and may reduce healthcare costs (36). Based on the evidence from the current study and on the inconsistencies found in previous studies, it is not recommended to mainly focus these interventions on lower socioeconomic groups. Intervention strategies may be developed for community-dwelling older adults in general, and do not have to be SEP-specific. However, still more evidence is needed from longitudinal studies, to see whether SEP is related to developing malnutrition over time. This may be addressed in future research, as well as comparisons between countries and settings.

In conclusion, this study did not provide convincing evidence for socioeconomic inequalities in malnutrition among older adults. In adjusted models, educational level and occupational level were not associated with nutritional risk. Some sub-domains of the MNAÂ®-SF were associated with SEP, but these associations were not consistent across socioeconomic indicators. Because of the severe consequences of malnutrition in terms of adverse health events and increased healthcare costs (36), it is important to develop interventions aimed at the prevention of malnutrition or at improving nutritional status. The findings of the current study suggest that these interventions do not have to be SEP specific. Additionally, nutritional screening should be performed for all older people, as malnutrition is a condition that can appear in any older adult, regardless of their socioeconomic group.

*Acknowledgments/Funding:* The FRADEA study was supported by the Castilla- La Mancha Health Research Foundation (FISCAM) [grant number Pi2006/42], and CIBERFES, Instituto de Salud Carlos III, Ministerio de EconomÃ­a y Competitividad, EspaÃ±a (Ayuda cofinanciada por el Fondo Europeo de Desarrollo Regional FEDER Una Manera de hacer Europa). Emiel O. Hoogendijk was supported by an NWO/ZonMw Veni fellowship [grant number 91618067]. Elsa Dent was supported by a National Health and Medical Council (NHMRC) Early Career fellowship [grant number APP1112672]. The funders had no role in the design or publication of the manuscript.
